# Interpretation of postmortem head computed tomography for non-traumatic in-hospital deaths by non-radiologists: a preliminary study

**DOI:** 10.1186/s40064-016-2653-z

**Published:** 2016-07-04

**Authors:** Asuka Araki, Noriyoshi Ishikawa, Saki Takami, Nahoko Ishikawa, Chika Amano, Haruo Takeshita, Riruke Maruyama

**Affiliations:** Department of Organ Pathology, Shimane University School of Medicine, 89-1 Enya, Izumo, Shimane 693-8501 Japan; Department of Pathology, Matsue Red Cross Hospital, 200 Horomachi, Matsue, Shimane 690-8506 Japan; Autopsy Imaging Center, Shimane University Hospital, 89-1 Enya, Izumo, Shimane 693-8501 Japan

**Keywords:** Postmortem computed tomography, Head, Non-radiologist, In-hospital death, Autopsy

## Abstract

**Purpose:**

Postmortem computed tomography (PMCT) has recently become important to clarify the cause of death in forensic medicine. It has also been proven to be useful for in-hospital deaths to a certain extent when interpreted by radiologists. However, accuracy of the interpretations of PMCT by non-radiologists remains to be elucidated. Nevertheless, they are often required to write death certificates based on the findings of PMCT in the absence of radiologists in Japan. We compared the interpretations of postmortem head CT (PMCT-H) by non-radiologists with the autopsy findings.

**Methods:**

This study included 13 patients who underwent both brain dissection at autopsy and PMCT between June 2011 and December 2014. All cases were non-traumatic in-hospital deaths. Interpretation of PMCT was performed by the clinicians in charge of the patients, not by radiology experts.

**Results:**

The patients were first examined with PMCT and then autopsies were performed. Ten out of 13 cases were confirmed to have no lesions in the cranial cavity by both PMCT-H and autopsy. Two cases were diagnosed with intracranial hemorrhage (intracerebral and/or subarachnoid hemorrhage) and one with recurrent malignant lymphoma by both the clinicians and the pathologists. Intracranial hemorrhages were thought to be the direct causes of mortality of the two patients, and recurrent malignant lymphoma was considered to be one of the cardinal findings of the cancer death. There were no discrepancies between PMCT-H and autopsy findings.

**Conclusions:**

The interpretations of PMCT-H by non-radiologists were completely the same as the autopsy findings regarding the non-traumatic in-hospital deaths in this study. It is premature to draw a definitive conclusion at present, but PMCT-H might be as effective as autopsy not only for those lesions described above but also for no remarkable changes in the brain. There has been no report on such a comparison. We believe further verification of the validity of interpretation of PMCT by non-radiologists is worthwhile in terms of death certificates made out in the absence of radiology experts and pathologists.

## Background

In accordance with decreasing autopsy rate in almost every country, the postmortem imaging has gradually become important as an alternative to autopsy in elucidating the cause of death. In Japan, since Ezawa et al. (2003) advocated autopsy imaging (Ai) as the term equivalent to postmortem imaging, it has been prevailing rapidly. Japan has overwhelmingly the highest number of CT scanners per capita in the world (almost three times the number in the United States in 2009) (OECD 2011) and clinicians can easily utilize them as a postmortem imaging device. However, compared to prevalence of CT scanners, the number of radiologists is insufficient (lowest among OECD countries) (Nakajima et al. 2008). Many clinicians in Japan often encounter situations where no radiologists are available, and if the cause of death is unclear but autopsy cannot be done, they have to interpret PMCT by themselves (Ikeda et al. 2009) before writing a death certificate. The role of postmortem imaging has been widely discussed in literature regarding non-violent adult deaths (Roberts et al. 2003; Weustink et al. 2009; Patriquin et al. 2001; Ros et al. 1990; Thali et al. 2003). One large study showed the accuracy of postmortem imaging as compared with autopsy by meticulous method (Roberts et al. 2012). In recent years, even PMCT-angiography (PMCTA) has been in use as to enhance the quality of PMCT (Busardò et al. 2015). However, such expensive methods are unlikely to apply to practical settings in small to middle-sized hospitals where the radiology workforce is insufficient. Furthermore, we must emphasize that the pathology workforce in Japan is also insufficient as compared with other countries: almost one-fifth of that of the USA per capita (The Japanese Society of Pathology 2003).

In order to understand and record the postmortem conditions of patients, almost all inpatients that died in Shimane University Hospital have been examined with unenhanced PMCT since June 2011. Taking advantage of this situation, we are currently elucidating the accuracy of postmortem imaging for non-traumatic in-hospital deaths interpreted by non-radiologists. In this report, the present study focused only on PMCT-H and radiological diagnoses made by clinicians who are not radiology experts because cranial lesions are very often associated with cause of death. Furthermore, even if autopsy is done, head dissection is frequently omitted because of family objection in non-forensic cases. Hence, it is important to interpret the PMCT-H accurately. Although the case number in the present study is small due to the decrease in the number of autopsies with head dissection, we believe it is worthwhile to report the results so far.

## Methods

### Study group

The average number of deceased patients in Shimane University Hospital during the study period was 370, and the mean autopsy rate and PMCT executing rate were 9.1 and 96.5 %, respectively. Thirteen cases including both head dissection and PMCT-H between June 2011 and December 2014 were drawn from the database, and all of them were adults and non-traumatic deaths. There was no suspected malpractice.

### Procedures

All PMCT studies were performed on a 16-multi detector row CT (Aquilion16; Toshiba Medical Systems Corporation, Tokyo, Japan) without contrast medium. The patients were laid in the supine position with arms adjacent to the body. The scanned area was between the head and lower legs. Contiguous 5 mm axial images were taken with reconstruction algorithm (soft tissue, lung, and bone), and around 1200 images were obtained per patient. Following PMCT, whole body autopsy was performed immediately, tissues were retained for histology and the photographic record of the macroscopic pathology was obtained from every subject. All cadavers underwent PMCT within 10 h of death. Only early postmortem changes (e.g. hypostasis) were observed, and late postmortem changes (e.g. decomposition) did not occur. Clinician interpretations of PMCT were drawn from our medical database in nine cases and inquiring survey from attending physicians in four cases. The CT scan images of every patient were interpreted by a group of two or more attending doctors. All but one group of attending doctors were internists: respiratory, hematological, neurological, rheumatological and cardiovascular doctors. The attending doctors for the case 4 were neurosurgeons. Their experience in reading CT scans varies from 3 to 10 years. Since detailed analysis and detection of the lesions not contributing to death were not the purpose of PMCT (e.g. evaluation of degenerative diseases such as Alzheimer’s disease), we adopted only findings considered to be the cause(s) of death with both PMCT and autopsy. Inconsequential changes found out by autopsy (e.g. ischemic changes due to agonal stage) were excluded.

### Ethics statement

Written informed consent was obtained from each patient before admission. The study was approved by the ethical committees of Shimane University Hospital (approved #: 1371).

## Results

**Fig. 1:**
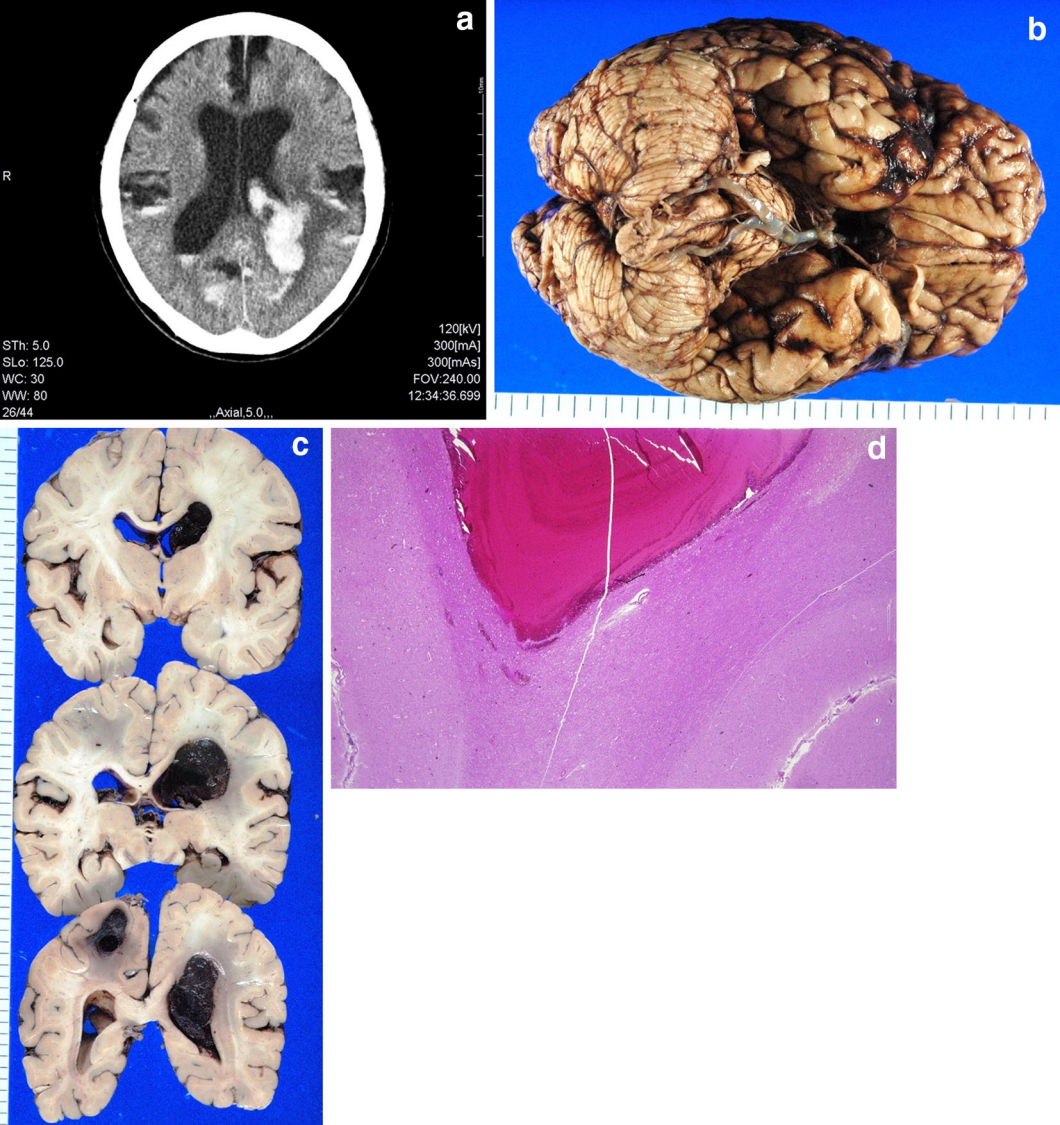
A 77 year-old-female who died of cerebral and subarachnoid hemorrhage (case no. 4). **a** Postmortem CT image through the brain showing cerebral hemorrhage with ventricular rupture and subarachnoid hemorrhage. **b** Macroscopic view of lower brain with subarachnoid hemorrhage. **c** Macroscopic view of a brain slice with cerebral and intraventricular hemorrhage. **d** Microscopic view of cerebral hemorrhage (hematoxylin and eosin)

**Fig. 2:**
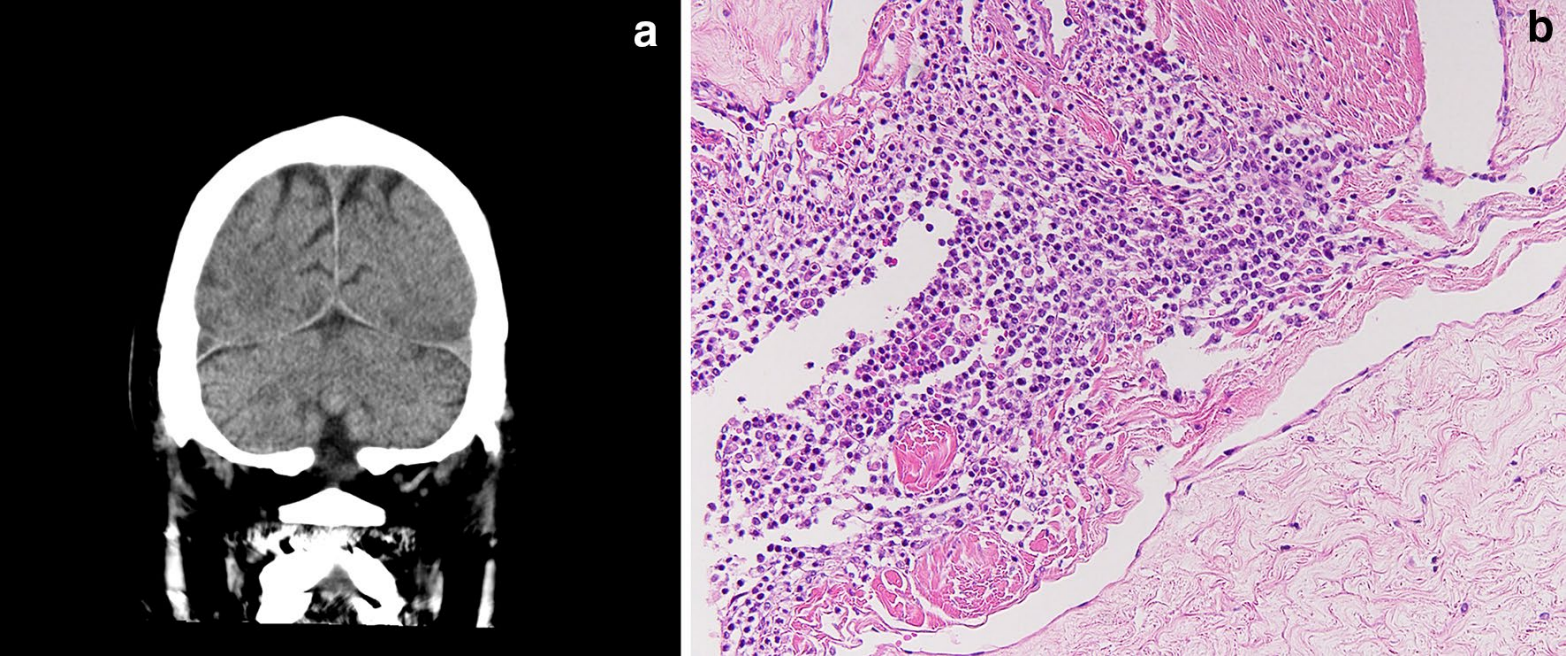
A 77 year-old-male who died of recurrence of malignant lymphoma of diffuse large B-cell type (case no. 13). **a** Postmortem CT image through the brain showing low density area in the superior sagittal sinus and right transverse sinus, indicative of a space-occupying lesion. **b** Microscopic view of the sagittal sinus showing lymphoma cell infiltration

Table 1 shows the summary of this study. Ten cases had no significant lesions except for minute changes not relevant to the cause of death in the cranial cavity by both PMCT-H and autopsy. Two cases were found to have intracranial hemorrhage by both modalities. The intracranial hemorrhage of case No. 4 and case No. 9 were attributed to bleeding tendency due to disseminated intravascular coagulation and rupture of an aneurysm of the anterior cerebral artery, respectively. These were the causes of death according to both clinicians (non-radiologists) and pathologists (Fig. 1). One case (No. 13) had intracranial recurrence of known diffuse large B cell type malignant lymphoma, which was thought to have played an important role in cancer death (Fig. 2). There were no discrepancies between clinicians’ PMCT-H assessments and autopsy findings.

**Table 1:** Summary of the patients enrolled with their PMCT and autopsy findings

Case	Age	Sex	Cause of death	PMCT-H findings	Autopsy findings of brain
1	29	M	Heart failure	NRC	NRC
2	59	F	Respiratory failure	NRC	NRC
3	77	M	Multiple organ failure	NRC	NRC
4	77	F	Cerebral and subarachnoid hemorrhage	Cerebral and subarachnoid hemorrhage	Cerebral and subarachnoid hemorrhage
5	79	M	Systemic fungal infection	NRC	NRC
6	87	M	Respiratory failure	NRC	NRC
7	74	M	Respiratory failure	NRC	NRC
8	68	M	Cancer death (lung carcinoma)	NRC	NRC
9	51	F	Subarachnoid hemorrhage	Subarachnoid hemorrhage	Subarachnoid hemorrhage
10	72	M	Unknown (sudden death)	NRC	NRC
11	73	M	Renal failure	NRC	NRC
12	78	M	Cancer death (pancreatic carcinoma)	NRC	NRC
13	77	M	Cancer death (malignant lymphoma)	Lymphoma cell infiltration	Lymphoma cell infiltration

*NRC* No remarkable changes

## Discussion

Takahashi et al. (2012) reported that fatal findings related to cause of non-traumatic death were detected by PMCT in 38.1 % of the patients who died in the emergency department in Japan. However, recent studies showed higher figures (around 70 %) in other countries (Westphal et al. 2012; Owais et al. 2010). These disparities are due to different factors that could affect the results such as cases enrolled (forensic vs. in-hospital deaths), interpreters involved (radiologists vs. non-radiologists), imaging techniques used (axial images only vs. multi-planar reconstruction or even three-dimensional images), and so on.

The present study showed complete concordance between the interpretations of PMCT–H and the autopsy findings. One of the reasons for this result is that CT excels in detection of hemorrhage: two of the three cases with head lesions had intracranial hemorrhage. Furthermore, the number of patients in the present study was only 13 due to the decreased rate of autopsies with head dissection in our hospital. Thus, the result is seemingly of limited value. However, we would like to emphasize that PMCT-H interpreted by non-radiologists proved to be accurate not only for hemorrhagic and neoplastic lesions, but also for no cranial lesions. Ideally, PMCT should be interpreted by radiologists, especially by those who are familiar with postmortem phenomena. Recently, more sophisticated methods like PMCTA and postmortem MRI have been in use. However, they are not always available in practical settings. Clinicians often have no choice but to assess the presence or absence of lesions employing PMCT without the aid of radiologists when the cause of death is unclear, unless autopsy including head dissection is done. This is not uncommon, at least in Japan, when clinicians are required to write a certificate of death. Furthermore, head dissection is frequently omitted in autopsy practices, as it is difficult to get consent from families.

In summary, PMCT-H findings of non-traumatic in-hospital deaths were assessed by non-radiologists, and they were as accurate as autopsy findings in the present study. It is premature to draw a definitive conclusion, but we believe it is worthwhile verifying the validity of non-radiologists’ PMCT interpretations as an alternative to autopsy when death certificates must be written in the absence of radiologists and pathologists. To the best of our knowledge, there have been no such reports. Further comparison between PMCT-H or even whole body PMCT findings assessed by non-radiologists and autopsy findings is mandatory, and it is currently underway in our institution.

## References

[CR1] Busardò FP, Frati P, Guglielmi G, Grilli G, Pinto A, Rotondo A, Panebianco V, Fineschi V (2015). Postmortem computed tomography and postmortem computed tomography–angiography: a focused update. Radiol Med.

[CR2] Ezawa H, Yoneyama R, Kandatsu S, Yoshikawa K, Tsujii H, Harigaya K (2003). Introduction of autopsy imaging redefines the concept of autopsy: 37 cases of clinical experience. Pathol Int.

[CR3] Ikeda N, Inakura M, Ishihara S, Ezawa H, Sakamoto T, Tamura S et al (2009) Investigative commission of autopsy imaging utilization. Ministry of Health, Labor and Welfare, Tokyo. http://www.mhlw.go.jp/stf/shingi/2r9852000000c011-att/2r9852000000c03j.pdf. Accessed 15 Sept 2013

[CR4] Nakajima Y, Yamada K, Imamura K, Kobayashi K (2008). Radiologist supply and workload: international comparison—Working Group of Japanese College of Radiology. Radiat Med.

[CR5] OECD (2011) Medical technologies. In: OECD, Health at a glance 2011: OECD indicators. OECD iLibrary, Paris. doi:10.1787/health_glance-2011-30-en

[CR6] Owais AE, Wilson TR, Khan SA, Jaidev J, Renwick I, Mitchell C, Macfie J (2010). Could pre-mortem computerized tomography scans reduce the need for coroner’s post-mortem examinations?. Ann R Coll Surg Engl.

[CR7] Patriquin L, Kassarjian A, O’Brien M, Andry C, Eustace S (2001). Postmortem whole-body magnetic resonance imaging as an adjunct to the autopsy: preliminary clinical experience. J Magn Reson Imaging.

[CR8] Roberts IS, Benbow EW, Bisset R, Jenkins JP, Lee SH, Reid H, Jackson A (2003). Accuracy of magnetic resonance imaging in determining cause of sudden death in adults: comparison with conventional autopsy. Histopathology.

[CR9] Roberts IS, Benamore RE, Benbow EW, Less SH, Harris JN, Jackson A, Mallett S, Patankar T, Peebles C, Roobottom C, Traill ZC (2012). Post-mortem imaging as an alternative to autopsy in the diagnosis of adult deaths: a validation study. Lancet.

[CR10] Ros PR, Li KC, Vo P, Baer H, Staab EV (1990). Pre-autopsy magnetic resonance imaging: initial experience. Magn Reson Imaging.

[CR11] Takahashi N, Higuchi T, Shiotani M, Hirose Y, Shibuya H, Yamanouchi H, Hashidate H, Funayama K (2012). The effectiveness of postmortem multidetector computed tomography in the detection of fatal findings related to cause of non-traumatic death in the emergency department. Eur Radiol.

[CR12] Thali MJ, Yen K, Schweitzer W, Vock P, Boesch C, Ozdoba C, Schroth G, Ith M, Sonnenschein M, Doernhoefer T, Scheurer E, Plattner T, Dirnhofer R (2003). Virtopsy, a new imaging horizon in forensic pathology: virtual autopsy by postmortem multislice computed tomography (MSCT) and magnetic resonance imaging (MRI)—a feasibility study. J Forensic Sci.

[CR13] The Japanese Society of Pathology (2003) http://shahojsp.umin.jp/information/us%20jpn%20hikaku%202003new.pdf. Accessed 13 Jan 2016

[CR14] Westphal SE, Apitzsch J, Penzkofer T, Mahnken AH, Knüchel R (2012). Virtual CT autopsy in clinical pathology: feasibility in clinical autopsies. Virchows Arch.

[CR15] Weustink AC, Hunink MGM, van Dijke CF, Renken NS, Krestin GP, Oosterhuis JW (2009). Minimally invasive autopsy: an alternative to conventional autopsy?. Radiology.

